# Source-sink behavioural dynamics limit institutional evolution in a group-structured society

**DOI:** 10.1098/rsos.211743

**Published:** 2022-03-23

**Authors:** Laurent Hébert-Dufresne, Timothy M. Waring, Guillaume St-Onge, Meredith T. Niles, Laura Kati Corlew, Matthew P. Dube, Stephanie J. Miller, Nicholas J. Gotelli, Brian J. McGill

**Affiliations:** ^1^ Department of Computer Science, University of Vermont, Burlington VT, USA; ^2^ Vermont Complex Systems Center, University of Vermont, Burlington VT, USA; ^3^ Department of Nutrition and Food Sciences, University of Vermont, Burlington VT, USA; ^4^ Department of Biology, University of Vermont, Burlington VT, USA; ^5^ Département de physique, de génie physique et d'optique, Université Laval, Québec (Québec), Canada G1V 0A6; ^6^ School of Economics, University of Maine, Orono ME, USA; ^7^ Mitchell Center for Sustainability Solutions, University of Maine, Orono ME, USA; ^8^ Mitchell Center for Sustainability Solutions, University of Maine, Orono ME, USA; ^9^ Centre interdisciplinaire en modélisation mathématique, Université Laval, Québec (Québec), Canada G1V 0A6; ^10^ Department of Social Science, University of Maine at Augusta, Bangor ME, USA; ^11^ Department of Computer Information Systems, University of Maine at Augusta, Bangor ME, USA

**Keywords:** source-sink dynamics, institutions, behavioural diffusion, cultural evolution, cooperation

## Abstract

Social change in any society entails changes in both behaviours and institutions. We model a group-structured society in which the transmission of individual behaviour occurs in parallel with the selection of group-level institutions. We consider a cooperative behaviour that generates collective benefits for groups but does not spread between individuals on its own. Groups exhibit institutions that increase the diffusion of the behaviour within the group, but also incur a group cost. Groups adopt institutions in proportion to their fitness. Finally, the behaviour may also spread globally. We find that behaviour and institutions can be mutually reinforcing. But the model also generates behavioural source-sink dynamics when behaviour generated in institutionalized groups spreads to non-institutionalized groups and boosts their fitness. Consequently, the global diffusion of group-beneficial behaviour creates a pattern of institutional free-riding that limits the evolution of group-beneficial institutions. Our model suggests that, in a group-structured society, large-scale beneficial social change can be best achieved when the relevant behaviour and institutions remain correlated.

## 1. Introduction

There is broad agreement that global crises such as climate change and pandemic response will require society to change. Therefore, a robust science of social change is needed. It has recently been suggested that the study of collective behaviour should be considered a crisis discipline [1]. Recent research, including the study of social tipping points [2], their impact on policy efficacy [3,4], and the feedbacks between policy and preferences [5], suggests a science of social change may be possible. But what constitutes social change? At a minimum, we suggest that social change must include both change in individual behaviours and change in institutions. Here, we study a model of social change that couples behavioural change with policy evolution.

The most difficult societal problems require both widespread change in individual behaviour and institutional change. But individual behaviour and group-level institutions are mutually interdependent, making solutions difficult to describe and achieve. On the one hand, large-scale behavioural change is slow or unpredictable unless supported by group-level institutions and policy. On the other hand, policy changes often follow from broad changes in individual preferences, beliefs or behaviour (e.g. gay marriage, civil rights). For example, no serious proposals for addressing climate change rely on the spread of voluntary individual behaviour alone; institutional support such as requirements and enforcement are clearly required. Yet without widespread individual support for climate policy, nations are unlikely to make institutional and structural change. This reciprocal causation makes addressing major societal goals particularly challenging because there the two most common approaches focus on only one aspect of the system. A policy-first approach uses a top-down change in institutions to affect large-scale behavioural change, for example by requiring and enforcing a behaviour (e.g. speeding fines, tax incentives, smoking bans). Alternatively a behaviour-first approach seeks to encourage voluntary behavioural change through bottom-up mechanisms such as unenforced behavioural standards (e.g. social distancing guidelines, voluntary participation in environmental programmes, and public service announcements generally). Both approaches have benefits and drawbacks.

**Figure 1.**
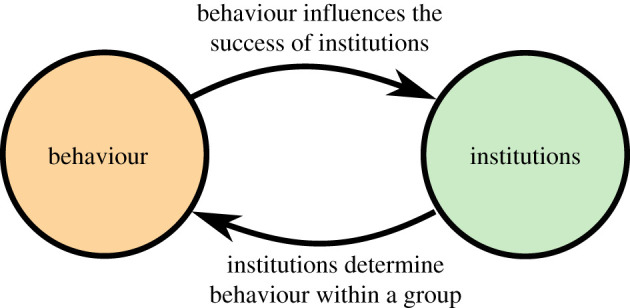
Individual behaviour and group institutions form a dynamical system with reciprocal causation. To understand the endogenous nature of social change, we must model the causal feedbacks between institutions and behaviour, and to influence social change we must be able to find and move towards beneficial states that are self-reinforcing.

Policy analysis is complex and policy outcomes are heavily determined by contextual factors. Policy design must therefore focus on local impacts, and thus often ignores where institutions come from, both in terms of how they originate via the policy process [6], and in terms of how policies spread via policy diffusion [7,8]. Institutions, governance, laws and rules emerge from the complex inter-individual process of cultural evolution and self-organization within human groups [9,10]. As a result, institutions are themselves distributions of individual cultural traits and behaviours [11]. However, research on the efficacy of policy options, e.g. [12–14], tends to be isolated from research on policy diffusion [7,15] and from research on the evolution of institutions [9,16–18]. Research that does link behavioural change to policy change typically focuses on only one half of the causal system. For example, a policy analyst might study how new COVID-19 policies determined individual mask-wearing behaviour. Conversely, a social scientist might study the spread of vaping behaviour through a social network and its impact on institutional responses. These are useful and necessary approaches; but behaviour and institutions are strongly reciprocally linked, as summarized in figure 1.

By contrast, a behaviour-first approach relies on the endogenous processes of behavioural adoption and transmission. Behaviour-first strategies, such as nudges [19] and viral advertising efforts, seek to leverage endogenous behavioural change for certain outcomes. Research on social tipping points shows that social momentum can be harnessed to improve well-being by boosting the spread of beneficial social norms and behaviours [2]. Similar research shows that these social tipping points can be caused by conformity and behavioural transmission and can create behavioural spillovers that determine the efficacy of policy [3,4,10]. When successful, behaviour-first approaches such as voluntary guidelines can lead to the diffusion of behaviour within communities. Research of this sort typically seeks to find a social tipping point past which the beneficial behaviour becomes ubiquitous [20]. If the behaviour becomes associated with values or social identities [21], the result can be the evolution of a self-reinforcing system of social norms: a new standard of behaviour [5,22]. However, behavioural diffusion and viral campaigns do not reliably generate social-tipping points, especially when the behaviour is costly.

A policy-first approach promotes a given behaviour via a change in institutions such as laws, rules or other formal group-level procedures. These institutional changes may simply be rules promoting or requiring behaviour, or they may entail behavioural support or enforcement. A policy approach can achieve rapid and large-scale behavioural change by altering the choices faced by an entire group. The scale and strength of the policy can determine the scale of adoption of the target behaviour to a great extent. However, incentives can undermine intrinsic motivation for the behaviour [23–25]. Moreover, behavioural requirements can be perceived as coercive even when they result from collective democratic policymaking, as has been evidenced with the COVID-19 pandemic [5]. Consequently, policy based on behavioural requirements can fail if people stop participating when the enforcement or incentive is withdrawn. Thus, policy-first solutions are often powerful but brittle.

One critical aspect of social change that is often overlooked are the feedbacks between individual behaviour and group-level policies [22,26]. There are multiple ways in which policies, whether voluntary or coercive, can cause self-defeating social dynamics among citizens and ‘crowd out’ beneficial behaviour. Those reactions depend upon the culture of the group, which in turn has been conditioned by previous policies and institutions [5]. Therefore, it is not possible to evaluate policy efficacy without understanding its reciprocal interactions with individual traits and behaviour.

We think it is useful to consider behavioural transmission and institutional change as entangled parts of a larger process of social change. Social change is attractive as a policy framework because it combines the benefits of endogenous (bottom-up) behavioural change and (top-down) interventions and policy, and allows us to ask when and how policy and behaviour come to be mutually reinforcing. However, unlike traditional policy frameworks, social change is at least partly an endogenous process, and cannot simply be implemented. In this paper, we ask how social change occurs when behaviours and institutions are mutually determined. We present a complex mathematical model for studying endogenous social change that couples simple mechanisms for the evolution of institutional policies and for the transmission of individual behaviours.

## 2. Linking behaviours and institutions

Behaviours and institutions are similar phenomena on different social scales; both are cultural traits. Behaviour, in most cases, is an individual cultural trait, or the expression of one, and institutions are group-level cultural traits. Both are invented, learned, modified, copied and re-transmitted, diffusing and evolving within a larger population. Thus, the simplest method to unify behavioural change and institutional change is to consider their transmission in parallel [18].

### 2.1. The transmission of behaviour

Behavioural diffusion has been studied from multiple perspectives, including the diffusion of innovation [27], behavioural contagion [28,29] and cultural transmission [30]. The transmission of cultural traits via social learning, imitation and teaching is central to the process of cultural evolution, and helps drive linguistic change, economic development and technological accumulation [31–33]. Other cultural traits including the language, preferences, identities, beliefs and values that determine behaviour are also transmitted via social learning between individuals [34]. This social science is complemented by research in disease modelling on behaviours that respond to, and spread with, an infectious disease [35,36].

Cooperative and pro-social behaviours are especially important, underlying successful group-level collective action by producing benefits beyond the actor. Here, we consider cooperative behaviour to be behaviour that carries group-level benefits. We are particularly interested in how institutions can promote the simple diffusion of such behaviours, which is motivated by previous results using a wealth of different approaches. Behavioural science shows that humans are conditional cooperators [37], cooperating when either the situation [38] or the social institutions support cooperative behaviour [39]. Moreover, cooperative and pro-social behaviour may sometimes be culturally transmitted between individuals within and between groups [40–42]. However, because cooperative behaviours often come at a cost to the actor, they rarely persist or spread on their own. The factors that promote the evolution of cooperation have also been heavily studied. These mechanisms have two common features: they are payoff-modifying in that they make cooperation less costly or non-cooperation more costly, and group-focused in that they operate within some group of individuals somehow defined. Cooperation stabilizing factors include decentralized individual-level mechanisms to exclude non-cooperators (e.g. ostracism) or reduce their payoffs (e.g. peer punishment), and centralized group-level institutions (e.g. pooled-punishment, or policing) which also change payoffs.

Since cooperation grows best in groups, inter-group processes such as competition and selection typically spur the evolution of cooperation in certain regards. Cooperation and institutional traits that support it can spread when human groups compete [18,43–45]. For example, trust tends to grow to higher levels in more competitive industries [46]. Game theoretic and evolutionary models show how cooperation can spread through group-level selection even when individuals face a social dilemma with other group members [47–49]. Analysis of these models reveals that a certain critical threshold of group-level pressure is required to make cooperation evolve generally. This cultural group selection mechanism helps explain both the spread of cooperation and the emergence of institutions that support it [18,26,48,50].

### 2.2. The evolution of institutions

Institutions, including centralized mechanisms for punishment, redistribution, division of labour and collective decision-making are a type of group-level cultural trait [11]. In comparison to decentralized behavioural regimes such as peer punishment or ostracism, institutions are widely considered the most effective and important mechanisms by which human groups stabilize cooperative behaviour. Institutions can stabilize cooperation and drive widespread behavioural adoption because they can render a cooperative behaviour individually beneficial by changing the outcomes and payoffs from each behavioural option. Institutional evolution is therefore believed to gradually increase the functionality of institutional arrangements for the groups that adopt them. For example, the most studied institutional features, Ostrom’s (1990) institutional rules [51], are considered to be group-beneficial cultural adaptations which evolved in large part to help groups maintain cooperation in important domains of shared interest [52]. Thus it is believed that institutions emerge and spread largely because they enable groups to resolve collective action problem and social dilemmas [33,44]. High levels of institutionalization, such as are found in rich industrial societies, can support a cooperative and productive economic system.

Institutions also spread between groups, via processes largely parallel to the mechanisms of social learning at the individual level. Research on policy diffusion shows that the spread of policy, rules and institutions between groups is also an important determinant of change [15,53]. Institutions may spread between companies, nations, municipalities, sports teams, or in any population of human groups. So, naturally, institutional evolution depends on the transmission of institutions between groups [9]. Thus, when organizations, nations and companies may imitate, modify and re-transmit helpful institutions, the result is the evolution of organizational structure, business models, municipal rules and national law. In fact, the concurrent spread of behaviour change and institutional support was recently identified as a needed avenue of research in public health modelling [54]. But currently, the transmission of individual-level behaviour and group-level institutions are typically considered in isolation.

Therefore, the relationship between the diffusion of behaviour and the spread of group policies and institutions is important for societal outcomes, yet remains understudied. On one hand, cooperative behaviour only tends to spread when institutions support it and render it less costly or altruistic for participants. On the other, institutions themselves only emerge when a group has a shared identity and intention to resolve a common goal: when cooperation is not a problem. So, the cooperation-institutions issue is a chicken-and-egg question; to understand one, we must understand both.

To help consider how policy and behaviour can produce beneficial societal outcomes, we ask how behaviour and institutions interact in a group-structured population. We develop a model of the coupled diffusion of individual behaviour and group-level institutions using a novel approach that combines epidemiological (e.g. [55]) and evolutionary approaches (e.g. [50]). We are interested in a group-beneficial behaviour, such as a cooperative or pro-social behaviour which will tend to dissipate without group-level support, but that can spread when a group adopts a policy to promote it. As our goal is to explain social change, not cooperation *per se*, we do not model individual costs and benefits in either evolutionary or economic terms, nor do we include utility maximization or payoff-biased imitation. We only model a group-beneficial behaviour that diffuses poorly on its own. The greater the group’s efforts to promote the behaviour, the more rapidly it spreads within that group. We couple the diffusion of this behaviour with a process of group-level institutional evolution, in which groups with more of the behaviour achieve better group-level outcomes. Groups with better outcomes are preferentially imitated, leading to the spread of institutions. We explore how this coupled system of behavioural and institutional evolution operates as a whole using a model that combines epidemiological spread of individual behaviour with group-level selection of institutions. Model code in C++ is available https://github.com/LaurentHebert/group-cultural-adaptation.

## 3. Model

We assume a population of size *N* ≫ 1, divided into *M* groups of equal size *n* = *N*/*M*. Groups contain *i* adopters of the behaviour and (*n* − *i*) non-adopters. The natural diffusion rate, *β*, is the rate at which the behaviour spreads from adopters to non-adopters within groups at a basic institutional level that allows but neither promotes nor discourages the behaviour. Adopters also lose that behavioural trait at a rate *γ*. Groups adopt an institution from a series of discrete levels of institutional strength, ℓ, which modify the rate of behavioural diffusion to be ℓ*β*. Note that without group-level institutions—i.e. at ℓ = 0—the behaviour does not spread at all. Therefore, like behaviours that benefit the group but incur individual costs such as cooperation and altruism, the behaviour we model will dissipate if not also adopted by one’s social contacts. The institutional strength of a group can therefore be conceptualized as setting the susceptibility of individuals in that group to adopting the behaviour. Finally, global behavioural diffusion, *ρ*, allows the behaviour to spread between groups, based on the institutional strength of the receiving group.

We then implement this system of behavioural diffusion within and among groups through a set of master equations [56] that tracks the fraction of groups *G*_*i*,ℓ_(*t*) with *i* adopters and institutional level ℓ. Omitting the time dependency for simplicity, the behaviour diffuses following:

3.1 $$\begin{array}{rl} \frac{d}{dt}G_{i,\ell }^{\mathrm{diff}} & =\ell \beta [(i-1)+R](n-i+1)G_{i-1,\ell }\\ & \;-\ell \beta (i+R)(n-i)G_{i,\ell }\\ & \;+\gamma (i+1)G_{i+1,\ell }-\gamma iG_{i,\ell }. \end{array}$$

In this equation, the first term corresponds to diffusion events bringing groups from state (*i* − 1, ℓ) to state (*i*, ℓ), which occurs proportionally to the internal diffusion rate ℓ*β* times the number of non-adopters *n* − (*i* − 1) who could adopt the behaviour. The factor in square brackets corresponds to the number of all adopters with influence over the non-adopters within that group, which includes *i* − 1 adopters within the group and the non-adopters in other groups who are exposed via global diffusion, i.e.

3.2 $$R=\rho \sum_{i^{\prime },\ell^{\prime }}i^{\prime }G_{i^{\prime },\ell^{\prime }},$$

where primes simply denote variables over which we sum to calculate a global quantity.

The second term in the master equation also corresponds to diffusion events but now taking groups out of state (*i*, ℓ) and into state (*i* + 1, ℓ). Finally, the last two terms correspond to relaxation events where adopters revert to their old behaviour; bringing groups from state (*i* + 1, ℓ) to (*i*, ℓ) or from (*i*, ℓ) to (*i* − 1, ℓ).

Additionally, we model institutional evolution among groups. Institutions are defined as a series of discrete levels of institutional strength ℓ ∈ [ℓ_min_, ℓ_max_], here set as ℓ ∈ [0, 5] but easily generalizable. Greater levels of institutional strength come with greater efficacy in promoting the group-beneficial behaviour (i.e. ℓ*β*) and with greater effort. Groups pay a cost *c* for each additional level of institutional strength, and gain a collective benefit *b* for each adopter in the group. Institutions evolve via a group selection process in which groups select their institutional strategy based upon the payoffs to groups with other strategies. We assume that a given level of institutional strength ℓ has a perceived fitness *Z*_ℓ_ given by

3.3 $$Z_{\ell }=\frac{\sum_{i^{\prime }}\exp(bi^{\prime }-c\ell )G_{i^{\prime },\ell }}{\sum_{i^{\prime }}G_{i^{\prime },\ell }},$$

where the denominator acts as a normalization factor. Note that only the fitness of a group’s strategy is directly observed and not the individual behaviour of its members. Based on this perceived fitness, groups can change their institutional strength following a second master equation,

3.4 $$\begin{array}{rl} \frac{d}{dt}G_{i,\ell }^{\mathrm{select}} & =\rho [G_{i,\ell -1}(Z_{\ell }Z_{\ell -1}^{-1}+\mu )+G_{i,\ell +1}(Z_{\ell }Z_{\ell +1}^{-1}+\mu )]\\ & \;-\rho (Z_{\ell -1}Z_{\ell }^{-1}+Z_{\ell +1}Z_{\ell }^{-1}+2\mu )G_{i,\ell }\;. \end{array}$$

These terms track flows of groups from institutional strength ℓ to ℓ + 1 and ℓ − 1 (and vice versa). These flows are assumed to occur proportionally to the relative fitness of different levels (e.g. a group moves from ℓ to ℓ + 1 proportionally to *Z*_ℓ+1_/*Z*_ℓ_) and the rate of global behavioural diffusion *ρ*, which is equivalent for individual behaviour and group institutions. Importantly, we also add a constant rate of transition *μ* regardless of fitness which allows the invention of new institutional levels (e.g. discovery of unoccupied institutional strategies).

The total dynamics of our model is then given by a set of master equations,

3.5 $$\frac{d}{dt}G_{i,\ell }=\frac{d}{dt}G_{i,\ell }^{\mathrm{diff}}+\frac{d}{dt}G_{i,\ell }^{\mathrm{select}},$$

defined over *i* ∈ [0, *n*] and a set of integers for levels of institutional strength ℓ ∈ [ℓ_min_, ℓ_max_]. We can thus track the dynamics of our system by numerically integrating all equations starting from arbitrary initial conditions (constrained only by $\sum_{i,\ell }G_{i,\ell }=1$).

The system is sufficiently complex to rule out analytical descriptions of equilibria, but we explore stability conditions analytically in electronic supplementary material, appendix. We simulate systems with identical initial conditions, specifically a uniform distribution of groups across institutional levels and a binomial distribution of *i* with average of 1% adopters. Stable long-run values were recorded when *t* ≥ 10 000 and if the expected average difference Δ*i* in groups was less than 10^−10^ over a Δ*t* = 1.

## 4. Results

**Figure 2.**
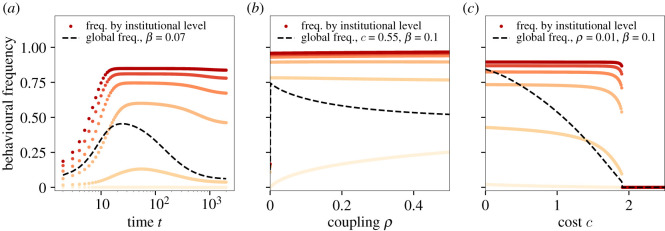
Frequency of behaviour in groups with different institutional strength. Within groups, the frequency of cooperative behaviour follows the strength of institutions, shown in shades of red (ℓ = 0 in light beige and ℓ = 5 in dark red). (*a*) The time dynamics of global behavioural frequency (black dashed line) and behaviour in groups can include patterns of surge and collapse. (*b*) Increasing global diffusion of behaviour, *ρ*, does not affect the frequency of behaviour in institutionalized groups but increases adoption of the behaviour in groups that have no institution (light beige). This shift increases the relative fitness of weaker institutions, leading to a decrease in global adoption of cooperation (figure 4). (*c*) Increasing the cost of institutions steadily decreases the global frequency of behaviour, eventually reaching an inflection point (here 1.9) above which increasing costs causes synchronized discontinuous collapse of cooperation across all groups. Other parameters are fixed at *n* = 20, *γ* = 1.0, *b* = 0.18 and *μ* = 10^−4^.

**Figure 3.**
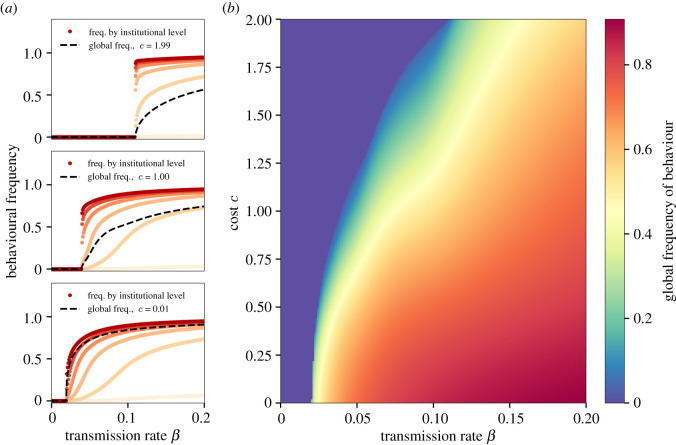
Behavioural transmission and institutional cost drive behaviour frequency. Institutional costs (*c* = 0.01, 1.0, 1.99) interact with behavioural transmission *β* to determine frequency of cooperation behaviour. (*a*) At lower costs behaviour emerges gradually with increasing transmission, but at higher costs this occurs more rapidly and uniformly across groups at different institutional levels. In groups with institutions ℓ ≥ 2, behaviour spreads extremely rapidly, while groups with ℓ = 1 see a slower and continuous activation with increasing *β*. Groups without institutions, ℓ = 0, displayed as the lightest shade, cannot generate cooperation but still see an increase in cooperation due to the global transmission from other groups. (*b*) This same interaction can be seen in the global frequency of behaviour across all combinations of *β* and *c*. Parameters fixed at *n* = 20, *γ* = 1.0, *b* = 0.18 and *μ* = 10^−4^.

**Figure 4.**
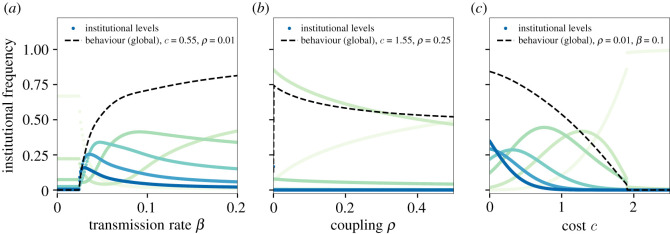
Institutional frequency among groups. The adoption of different levels of institution (i.e. $\sum_{i^{\prime }}G_{i^{\prime },\ell }$) is shown with ℓ = 0 in light green and ℓ = 5 in dark blue). (*a*) At low values of *β,* groups with ℓ = 0 are most common. As the transmission rate crosses the threshold, stronger institutions (ℓ > 2) proliferate rapidly, while weaker institutions first decline in frequency, then grow to become dominant. Institutions of ℓ = 2 or ℓ = 3 become most popular at intermediate values of *β*. Stronger institutions are progressively favoured at lower value of cost *c* and global diffusion *ρ*. (*b*) Increasing the global diffusion rate *ρ* confirms the emergent institutional free-riding problem: the no-institutions strategy progressively gains in popularity as global diffusion of cooperation increases, eventually becoming dominant. (*c*) Stronger institutions are favoured at lower cost. As institutional costs increase, lower levels of institution are progressively preferred. This dynamic continues until the lowest non-zero institutional level ℓ = 1 also loses its fitness at the cost threshold, where these groups experience a discontinuous transition. Parameters fixed at *n* = 20, *γ* = 1.0, *b* = 0.18 and *μ* = 10^−4^.

**Figure 5.**
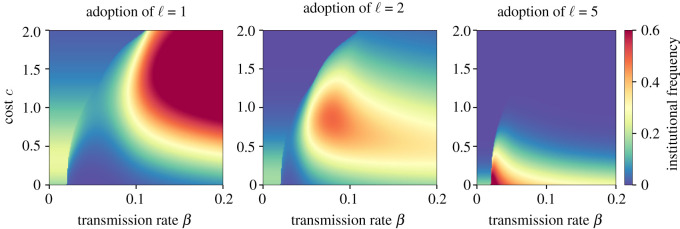
The prevalence of institutions of different strengths varies by costs and transmission rate. Panel replicates the numerical experiment of figure 3, but shows the adoption of institutions of level 1, 2 and 5, respectively. This comparison highlights the phenomenon of institutional localization in which a given institutional level dominates the fitness landscape in some subset of parameter space. This phenomenon is analytically explored in electronic supplementary material, appendix.

The model produces a set of coevolutionary dynamics between behaviours and institutions, some of which were unexpected, and hold value as tools for understanding social change. We summarize model behaviour in figures 2–5.

As expected, the model confirms that cultural group selection on institutions can drive evolution of cooperation or pro-social behaviour, as others have demonstrated [26,47,48]. We find that for behavioural-institutional coevolution to take off, two fundamental criteria must be met: institutional implementation costs, *c*, must be below a threshold value (see figure 2, and the rate of behavioural transmission within groups, *β*, must be above a critical threshold (figure 3). These thresholds are sharp. Even small changes in *c* and *β* can have catastrophic effects (figure 3). In fact, we find a rich mix of both continuous and discontinuous emergence of the behaviour depending on institutional levels and parameter values. We explore the mathematical conditions around these transitions in electronic supplementary material, appendix.

Qualitatively, no institutions are possible if institutional costs are too high, and the behaviour never spreads. Lowering institutional costs slightly below the threshold results in a sharp discontinuity such that institutions and behaviour both flourish. Further decreases do not change qualitative outcomes. A similar threshold also exists for within-group behavioural transmission, *β*. Unlike the cost threshold, however, increases in *β* above the threshold result in further spread of both behaviour and institutions. However, if costs are low, institutions may still evolve even with a very low *β* (figure 4*a*).

Naturally, simple institutions (i.e. ℓ = 1) emerge and become most common across a large range of parameters (*c*, *β*, *ρ*) (figure 4*c*). Stronger institutions (i.e. ℓ ≥ 2) also evolve if costs are sufficiently low (figure 4). However, as costs rise towards the threshold, the greatest level of institution that can be sustained declines steadily. Note that our model is harsh on the evolution of stronger institutions, because we do not allow institutions to modify costs, which they are typically assumed to do. Therefore, we suspect that the model underestimates institutional evolution.

Under certain conditions, the model exhibits an unexpected pattern of bi-modal institutional success. This occurs when a certain level of institutionalization is required to maintain the behaviour locally, but only some groups become self-sustaining. For example, close to the sustainability thresholds, it is possible to arrive at a state in which approximately 20% of groups have no institution, approximately 25% with level 3 institutions, but very few have level 1 or 2 institutions (figure 4*a*). This state emerges when the intervening institutional level (ℓ = 1 or 2) has lower fitness than either ℓ = 0 or ℓ = 3, hindering non-institutional groups from moving to stronger institutions. We call this phenomenon institutional localization. We explore it visually in figure 5 and analytically in details in electronic supplementary material, appendix.

The rate of global behavioural diffusion, *ρ*, also creates a counterintuitive effect. As *ρ* increases, total behavioural adoption and institutions both *decrease* (figure 4*b*). Increased global diffusion allows the cooperation to spread into groups that do not have institutions to generate or support it. Cooperation in receiving groups does not persist, but still increases their fitness. Higher fitness outcomes for poorly institutionalized groups then creates selection against stronger institutions. In essence, high between-group transmission, *ρ*, creates *behavioural source-sink dynamics*: behaviour generated in a group with institutional support diffuses to other groups, improving the outcomes of those groups without effective local institutions. Put in evolutionary terms, this ‘institutional free-riding’ caused by between-group behavioural transmission can halt cultural group selection and the evolution of group-beneficial institutions.

The source-sink dynamics of cooperation have an important influence on model outcomes. In the region around, the emergence threshold model behaviour is complex and outcomes are sensitive to initial conditions. In this region, we find non-monotonic time series in which behaviour and institutions can spread together for a time, only to collapse later (figure 2*a*). In certain parameter combinations, the model exhibits multiple stable equilibria, ending either in the collapse of behaviour and institutions (e.g. all groups in *i* = ℓ = 0) or with a steady-state non-zero level of both behaviour and institutions, depending finely on initial conditions. This model thus provide an interesting mathematical sandbox for future work considering how to design promotion of beneficial collective behaviour and institutional support.

## 5. Conclusion

Our model helps us understand social change when behaviour and institutions are mutually linked. Connecting the diffusion of group-beneficial behaviour to the spread of policy and supporting institutions reveals a set of social dynamics that are not often studied. Our analysis shows that group-beneficial behaviour can be subject to source-sink dynamics in which cooperation generated by groups with strong institutions spreads to less-institutionalized groups. These behavioural source-sink dynamics create an emergent pattern of institutional free-riding in which receiving groups benefit from the imported cooperative behaviour, but cooperation dies away in those groups without institutional support. Thus, receiving groups act as a cooperation sink. We also find that when the global spread of cooperation, *ρ*, is sufficiently strong, these dynamics can halt the evolution of institutions. These dynamics can dampen or halt cultural group selection for institutions when the behavioural diffusion increases the fitness of receiving groups with weak institutions.

Source-sink dynamics of cooperation may be influencing social evolution all the time, and examples abound. One area of interest is when the provision of public goods is difficult to understand or perceive. When the cooperation of one group creates benefits for other groups, institutional free-riding may result. One example of this may occur in national tax systems when tax revenue is shared between regions. If such revenue sharing enhances the perceived success of recipient states, it may also undermine institutional evolution among states. Another immediate example concerns the heterogeneous and slow spread of robust public health policy for fighting COVID-19 in the USA [57–59]. States with robust policy (i.e. masking, social distancing and vaccination requirements) probably spread those beneficial behaviours to states with weak public health policy. This behavioural spillover could artificially increase the actual or perceived success of states with weak policies, slowing the uptake of effective policies around the country.

Source-sink dynamics of cooperation might also help explain long-term patterns of social change, such as the decline of unions in the USA [60]. Collective bargaining often improves work conditions and pay for union members, but can also benefit non-union members. For example, in right-to-work states, people can reap the rewards of a union without joining [61]. This undercuts the strength of the local union, but it may also reduce union fitness generally, slowing their spread elsewhere. Thus, limiting negotiated benefits to members might improve the correlation between institution and behaviour, helping to maintain both. Another example comes from food safety regulations. A lack of awareness of the role of the safety regulations can lead municipalities to repeal them. But, when people mostly consume food from safely regulated production systems, those non-regulated municipalities are protected from negative consequences, and the reduced safety standards can spread. Something similar has been observed in Maine, where reduced food safety standards have spread between many small towns [62]. These examples suggest that policies that create public goods can be made more durable if they also make their supply of those public goods highly observable. Our impetus for this project is the category of societal or global-scale problems such as climate change which may require large-scale social change. We believe the source-sink dynamics of group-beneficial behaviour provide a new and useful framework and are worthy of additional study.

In combining behavioural transmission with institutional evolution, the model shows that it is possible to overcome limitations of the policy-first and behaviour-first approaches. Specifically, our model has implications for intervention efforts to achieve large societal goals that require social change which do not emerge from the standard approaches. First, we find that, despite reciprocal dynamics between behaviour and policy, the spread of institutions has a greater influence on behavioural diffusion than the reverse. This is an important insight for both social scientists and policymakers in the age of nudges and viral behavioural campaigns: as a group-level cultural trait, policy has an outsized influence on the evolution of a group-structured society. But social change is not owned and cannot be controlled by any one actor, even including legitimate governments. Any actor within society might look to influence social change. Indeed, beyond political domains many actors already intentionally and strategically influence social change. For example, for-profit corporations spend large sums of money in advertisements to modify consumer preferences and purchasing behaviour. Therefore, it is useful to provide intervention points for actors in different social positions. We focus on two levels, first the level of the group (such as a state or organization) and second, the level of the population of groups (such as a nation).

Group-level actors, such as state governments or organizations, have two major avenues for action that emerge from our framework. First, groups can work to improve the spread of the cooperative or beneficial behaviour within the group. For example, create meaningful rewards for the group-beneficial behaviour, or ways of celebrating the contributions of those who contribute. Second, groups can adopt new institutions (which enhance behavioural transmission) by learning from other innovative groups. Innovative institutions may be social in nature, such as finding and enhancing ways for individuals to reward, recognize and congratulate each other on their contributions. But groups should also be careful to avoid relying on externally generated cooperation, lest it cease to flow. This framework could be useful for economic development in rural states.

Population-level actors, such as national governments, can work to improve the spread of policies that support beneficial behaviour between groups. There are two main fronts in this effort. First, reducing the costs of implementing policies that support group-beneficial behaviour can be very effective at spreading both behaviour and policy. For example, a central government could provide financial support to cities that adopt a policy to support climate-friendly behaviour. Second, increasing between-group learning of policy options can accelerate behaviour-policy coevolution. For example, the US Federal Government could enhance the transmission of beneficial state policies by supporting conferences between state governments to facilitate the exchange of ideas and solutions in a domain of interest (e.g. combating addiction).

Actors at the population level must also contend with source-sink dynamics of behaviour which can undermine cooperation and institutional evolution. This process is determined in part by the rate of transmission of group-beneficial behaviour between groups. Therefore, our model suggests that efforts to spread cooperation globally are likely to be somewhat self-defeating. Furthermore, we would caution against strategies for reducing the transmission of cooperation between groups, as such policies could counteract the transmission of cooperation *within* groups, which is of even greater importance. Instead, population-level actors should work to create a pattern of reinforcement between group-beneficial behaviour and local institutions. One way to accomplish this is through correlated pilot projects: efforts to boost the *correlated* spread of beneficial behaviour and supporting institutions. In our model, behaviour and institutions spread best when they are correlated across groups and with beneficial outcomes. Therefore, policy approaches might focus on promoting strong pilot studies and local public trials with high chances of success to be copied elsewhere. Future research could use this model to study examples of pilot project diffusion. For example, a study of coastal management pilot projects in South Africa suggests that the coupled diffusion of cooperation and institutions may be useful for achieving societal transitions to more sustainable regimes [63]. It seems likely that similar dynamics might underlie the diffusion of environmental regulation [15,64,65].

Our approach can be improved and expanded in a number of useful directions. First, the current model ignores individual payoffs and decision-making. Adding these would bring it in line with the game theoretic and evolutionary literature on cooperation, and might have implications for the extent of source-sink dynamics of group-beneficial behaviour. For example, one could investigate new mechanisms to accelerate social transitions by mitigating institutional free-riding. We can envision modelling organizational or financial reporting policies as a mechanism through which the observed fitness of a group can now explicitly depend on their institutional level ℓ. This signalling would inform groups not only about successful strategies but the required context for them to be successful (e.g. groups should only increase their institutional level in synchrony with local behaviour, so that institutional changes reliably generate group-level benefits). The model could be made more evolutionary as well. It could be implemented as a full multi-level selection model with emergent pressures of selection operating at both individual and group levels [26]. Or, future iterations could specify the social learning mechanisms in operation at each level.

Our model has the potential to shed light on a set of social processes that may be very influential in social evolution. If the dynamics that we demonstrate here are as prevalent as they appear, the implications of this study are deep and far-reaching. Moreover, our model provides important insights on the efficacy of approaches for addressing global challenges such as climate change. It provides a novel framework to study intervention regardless of the social scales at which actors operate.

## Data Availability

Model simulation code is available in C++ at https://github.com/LaurentHebert/group-cultural-adaptation, and has been archived within the Zenodo repository: https://doi.org/10.5281/zenodo.5949710. Additional analyses are provided in electronic supplementary material [66].
